# Fetal Cortical Plate Segmentation Using Fully Convolutional Networks With Multiple Plane Aggregation

**DOI:** 10.3389/fnins.2020.591683

**Published:** 2020-12-02

**Authors:** Jinwoo Hong, Hyuk Jin Yun, Gilsoon Park, Seonggyu Kim, Cynthia T. Laurentys, Leticia C. Siqueira, Tomo Tarui, Caitlin K. Rollins, Cynthia M. Ortinau, P. Ellen Grant, Jong-Min Lee, Kiho Im

**Affiliations:** ^1^Department of Electronic Engineering, Hanyang University, Seoul, South Korea; ^2^Fetal-Neonatal Neuroimaging and Developmental Science Center, Boston Children’s Hospital, Harvard Medical School, Boston, MA, United States; ^3^Division of Newborn Medicine, Boston Children’s Hospital, Harvard Medical School, Boston, MA, United States; ^4^Department of Biomedical Engineering, Hanyang University, Seoul, South Korea; ^5^Mother Infant Research Institute, Tufts Medical Center, Tufts University School of Medicine, Boston, MA, United States; ^6^Department of Pediatrics, Tufts Medical Center, Tufts University School of Medicine, Boston, MA, United States; ^7^Department of Neurology, Boston Children’s Hospital, Harvard Medical School, Boston, MA, United States; ^8^Department of Pediatrics, Washington University in St. Louis, St. Louis, MO, United States

**Keywords:** deep learning, fetal brain, cortical plate, segmentation, hybrid loss, MRI

## Abstract

Fetal magnetic resonance imaging (MRI) has the potential to advance our understanding of human brain development by providing quantitative information of cortical plate (CP) development *in vivo*. However, for a reliable quantitative analysis of cortical volume and sulcal folding, accurate and automated segmentation of the CP is crucial. In this study, we propose a fully convolutional neural network for the automatic segmentation of the CP. We developed a novel hybrid loss function to improve the segmentation accuracy and adopted multi-view (axial, coronal, and sagittal) aggregation with a test-time augmentation method to reduce errors using three-dimensional (3D) information and multiple predictions. We evaluated our proposed method using the ten-fold cross-validation of 52 fetal brain MR images (22.9–31.4 weeks of gestation). The proposed method obtained Dice coefficients of 0.907 ± 0.027 and 0.906 ± 0.031 as well as a mean surface distance error of 0.182 ± 0.058 mm and 0.185 ± 0.069 mm for the left and right, respectively. In addition, the left and right CP volumes, surface area, and global mean curvature generated by automatic segmentation showed a high correlation with the values generated by manual segmentation (*R*^2^ > 0.941). We also demonstrated that the proposed hybrid loss function and the combination of multi-view aggregation and test-time augmentation significantly improved the CP segmentation accuracy. Our proposed segmentation method will be useful for the automatic and reliable quantification of the cortical structure in the fetal brain.

## Introduction

A fundamental method for understanding brain development and disease is the quantitative analysis of magnetic resonance imaging (MRI) data, which requires preprocessing steps such as brain extraction, tissue segmentation (gray matter, white matter, and cerebrospinal fluid), and specific region-of-interest segmentation. Advances in MRI technology have enabled *in vivo* human fetal MRI studies to examine early brain development during the prenatal period. Among several quantitative indices of the human fetal brain, cortical volume and cortical folding patterns are crucial to the characterization and detection of abnormal brain development (Scott et al., 2011; Clouchoux et al., 2012; Im et al., 2013, 2017; Tarui et al., 2018; Ortinau et al., 2019; Yun et al., 2020a). For a reliable and sensitive analysis of its volume and surface folding patterns, accurate segmentation of the cortical plate (CP) is necessary. However, manual or semi-automatic segmentation has been used in previous studies which is a highly time-consuming and challenging task with high inter- and intra-rater variability. In addition, because fetal brains exhibit dramatic changes in size, cortical shape, cellular compartments, and image contrast at tissue boundaries, which vary with gestational age (GA) compared to child or adult brains, previous methods that were developed for the cortical gray matter segmentation of mature brains are not applicable to fetal brain segmentation.

Over the past decade, several algorithms for automatic CP segmentation from fetal MRI have been proposed. The expectation-maximization (EM) algorithm and atlas-based segmentation method have been employed for fetal brain tissue segmentation (Bach Cuadra et al., 2009; Habas et al., 2010; Serag et al., 2012; Wright et al., 2014). However, previous studies have reported results from a narrow GA range in a small number of subjects, and/or exhibited large errors [4–16 subjects, accuracy of CP segmentation measured by Dice coefficient = 0.63–0.84 and mean surface distance (MSD) error = 0.70–0.86 mm] (Bach Cuadra et al., 2009; Habas et al., 2010; Serag et al., 2012; Wright et al., 2014). The EM algorithm requires the precise estimation of a mixture of tissue probability using linear and non-linear registration between target images and a brain atlas. Likewise, atlas-based segmentation requires precise registration, including a non-linear approach between the target image and brain atlas. Fetal CPs have a very thin band-shaped structure, and the boundary of CPs is ambiguous owing to a low effective MRI resolution and the partial volume effect, which limits the accuracy of registration. Therefore, it may be difficult to accurately extract thin CPs from fetal MRI using the EM algorithm and atlas-based segmentation.

Recently, deep learning in the field of image segmentation has shown superior performance compared to traditional methods such as EM algorithm. Among various deep learning algorithms, the convolutional neural network (CNN) has been widely used for brain tissue and region segmentation in postnatal MRI data (Zhang et al., 2015; Kleesiek et al., 2016; Milletari et al., 2016; Ghafoorian et al., 2017; Chen et al., 2018; Kushibar et al., 2018; Wachinger et al., 2018; Alom et al., 2019; Guha Roy et al., 2019). Fetal CP segmentation methods based on MRI and ultrasound have been proposed using CNN (Khalili et al., 2019; Dou et al., 2020; Wyburd et al., 2020). One peer-reviewed MRI study proposed fetal brain tissue segmentation using a two-dimensional (2D) semantic CNN model that can segment seven brain tissues, including the CP (Khalili et al., 2019). However, the authors trained a CNN using the basic Dice loss, which maximize the Dice coefficient of segmentation. The basic Dice loss may not be optimal for relatively small areas in the multi-label segmentation problem, which may be a reason for the low accuracy of CP segmentation (Sudre et al., 2017; Wong et al., 2018). They obtained a CP segmentation accuracy that was relatively lower than the overall average Dice coefficient (CP: Dice coefficient = 0.835; Overall: Dice coefficient = 0.892) with a small number of fetal brain MRIs for a wide range of GA (12 fetuses from 22.9 to 34.6 weeks). Moreover, 3D information of the brain structures was not fully utilized in their methods, since they trained the network model using only coronal slices. To overcome the limitations in the previous methods, we propose an enhanced method for the automatic segmentation of the fetal CP using deep learning based on a large dataset of fetal brain MRIs. Our proposed method is focused on CP segmentation as our aim is to achieve the optimal accuracy of cortical volumes and surfaces. Numerous segmentation labels may require the complicated deep learning network and achieve inaccurate performance of CP segmentation. We propose a novel hybrid loss function and utilize a multi-view aggregation with test-time augmentation (MVT) approach to enhance the performance of CP segmentation. We adopt a focal Dice loss function, which is an exponential logarithmic Dice loss, to assign a large gradient to the less accurate labels (Wong et al., 2018). Our hybrid loss additionally includes a novel boundary Dice loss to accurately segment the CP boundary areas. In addition, the multi-view aggregation technique is used to enhance the segmentation accuracy by applying a 3D information to a 2D deep learning network. It combines three results from separate learning networks of 2D slices from three orthogonal planes (axial, coronal, and sagittal) to generate the final segmentation (Guha Roy et al., 2019; Jog et al., 2019; Estrada et al., 2020). The test-time augmentation (TTA) technique can obtain more robust prediction results using multiple predictions for a single input by applying the augmentation to test data, which is often used for the training phase in deep learning networks (Matsunaga et al., 2017; Jin et al., 2018). In this study, we applied both multi-view aggregation and TTA methods to obtain multiple results in each plane and to combine all results generated from the three planes. The hybrid loss was compared with the basic Dice loss, and MVT was compared with the results of the multi-view aggregation, TTA, and single view prediction. We hypothesized that MVT performs better than multi-view or TTA because it combines more segmentation results without changing the network and multi-view training structure. Furthermore, volume- and surface-based indices were extracted from both ground truth and automatic segmentation results and then compared to examine the reliability of brain measurements calculated from our segmentation.

## Materials and Methods

### Dataset

The use of fetal MRIs was approved by the Institutional Review Boards at the Boston Children’s Hospital (BCH) and Tufts Medical Center (TMC). Typically developing (TD) fetal MRIs were collected from subjects by recruitment, and retrospectively from clinical fetal MRIs performed to screen for abnormalities at BCH but found to be normal. Inclusion criteria for TD fetuses included no serious maternal medical conditions (nicotine or drug dependence, morbid obesity, cancer, diabetes, and gestational diabetes), maternal age between 18 and 45 years, fetal GA between 22 and 32 weeks GA. Exclusion criteria included multiple gestation pregnancies, dysmorphic features on ultrasound (US) examination, brain malformations, or brain lesions on US, other identified organ anomalies on US, known chromosomal abnormalities, known congenital infections and any abnormality on the fetal MRI. A total of 52 TD fetuses (22.9–31.4 weeks of pregnancy) were identified and used in this study. Fetal brain MRIs were acquired on a Siemens 3T Skyra scanner (BCH) or Phillips 1.5 T scanner (TMC) using a T2-weighted half-Fourier acquisition single-shot turbo spin-echo (HASTE) sequence with a 1-mm in-plane resolution, field of view (FOV) = 256 mm, TR = 1.5 s (BCH) or 12.5 s (TMC), TE = 120 ms (BCH) or 180 ms (TMC), and slice thickness = 2–4 mm. After localizing the fetal brain, the HASTE scans were acquired multiple times in different orthogonal orientations (a total of 3–10 scans) for reliable motion correction and the 3D reconstruction of fetal brain MRI.

### Preprocessing

First, we performed preprocessing on fetal brain MRIs (Im et al., 2017; Tarui et al., 2018; Yun et al., 2019, 2020b). Using multiple scans of HASTE, a slice-to-volume registration technique was adopted to combine 2D slices of fetal brain MRIs to create a motion-corrected 3D volume (Kuklisova-Murgasova et al., 2012). We set the resolution of the reconstructed volume to a 0.75-mm isotropic voxel size. Because the size, position, and orientation of the reconstructed volumes vary for different fetuses, the reconstructed volumes were linearly registered to a fetal brain template using “FLIRT” in FSL and transformed to a standard coordinate space (Jenkinson et al., 2002; Serag et al., 2012). Then, the CP volume and whole inner volume of the CP were semi-automatically segmented into left and right based on the voxel intensities by two trained raters, and they were manually modified to obtain the final segmentation by a single person. The final segmentation from the semi-automatic approach was used as ground truth.

We performed additional processes on the registered MRI for better segmentation performance. First, we removed unnecessary non-brain voxels from the registered volume by multiplying them by the brain mask of the template. Second, the *z*-transformation was applied to normalize the intensity distribution across the entire MRI scan. Finally, the scanned image was cropped based on the size of the dilated template brain mask and the size of the 2D image of each axis plane, unified to a 128 × 128 2D slice by zero padding.

### Network Architecture

**FIGURE 1:**
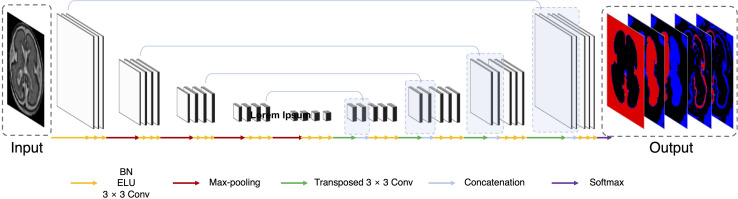
Illustration of proposed network based on U-Net. Our network uses a 128 × 128 2D slice as the input and predicts the probability of five labels (background, left and right CP, and left and right inner volume of CP).

The deep learning network architecture is shown in Figure 1. We configured the contracting (left side) path, expansive (right side) paths, and skip connections, similar to the U-Net (Ronneberger et al., 2015). The structure comprises repeated layers of the batch normalization (BN), exponential linear units (ELU), 3 × 3 zero-padded convolution, and a 2 × 2 max pooling with stride 2 (Ioffe and Szegedy, 2015; Clevert et al., 2016). Each network layer is divided into blocks based on the size of the feature map. Each block represents a structure in which the BN, ELU, and convolution layers are present in triplicate. The order of the layers in the block was composed of BN, ELU, and convolution by referring to the evaluation result of the previous study (He et al., 2016). Thirty-two feature maps were generated by convolution in the first block, and the number of feature maps doubled as the size of the block became smaller, finally generating 512 feature maps. In the expansive path, we extended the feature map of the lower feature map size block to the size of the higher size block using 3 × 3 transposed convolution. The extended feature map and the last feature map of a corresponding block on a contracting path of the same size were concatenated and used as inputs of repeated convolution. In the last layer, 1 × 1 convolution was employed to compress the desired number of labels from the 32 feature maps to 5 (including background), and softmax activation was applied to create a probability value for each label.

We additionally trained a 3D network to compare with the performance of the multi-view aggregation. The 3D network structure is basically the same with 2D network, and the 2D layers are simply changed to 3D layers (e.g., 2D convolution to 3D convolution). However, due to the limitation of the graphic processing unit (GPU) memory, the number of feature maps generated by convolution in the first block starts with eight, and the number of feature maps at the largest is 128.

### Loss Function

#### Dice Loss

The Dice loss function was introduced in a previous medical image segmentation study (Milletari et al., 2016). The authors calculated the Dice loss using the Dice coefficient, which is an index used to evaluate the segmentation performance. For segmentation of the prostrate, the Dice loss exhibited superior performance to the re-weighted logistic loss. In this study, the Dice loss (*L*_*Dice*_) was employed according to the following function:

$$L_{\text{Dice}}\,\left(g,p\right)=1-\frac{1}{N_{\text{l}}}\,\left(\sum_{\text{l}}\frac{2\left(\sum_{\text{i}}g_{\text{li}}p_{\text{li}}\right))+\epsilon }{\sum_{\text{i}}\left(g_{\text{li}}+p_{\text{li}}\right)+\epsilon }\right)$$

Here, *i* depicts the pixel location, *l* represents the label, and *N*_*l*_ is the total number of labels. *p*_*li*_ is the softmax probability calculated from the deep learning network, and *g*_*li*_ is the ground truth probability at location *i* and label *l*. ϵ is the smoothing term to prevent division by zero. The Dice coefficient of each label has a value between 0 and 1. The loss function (1– averaged Dice coefficient) is used for training.

#### Hybrid Loss

The Dice loss demonstrated its usefulness in the segmentation problem of medical images (Milletari et al., 2016; Guha Roy et al., 2019; Khalili et al., 2019). However, new losses that improve the Dice loss have recently been introduced (Sudre et al., 2017; Wong et al., 2018). The Dice loss is unfavorable for relatively small structures, as misclassifying a few pixels can lead to a large reduction in the coefficient (Wong et al., 2018). Therefore, we adopted the logarithmic Dice loss (focal loss; *L*_*focal*_), which focuses on less accurate labels (Wong et al., 2018):

$$L_{\text{focal}}\left(g,p\right)=\frac{1}{N_{\text{l}}}\left(\ln{\left(\sum_{\text{l}}\frac{2\left(\sum_{\text{i}}g_{\text{li}}p_{\text{li}}\right))+\epsilon }{\sum_{\text{i}}\left(g_{\text{li}}+p_{\text{li}}\right)+\epsilon }\right)}^{\gamma }\right)$$

Here, γ dictates the non-linearities of the loss function. In this study, the optimum value of γ was 0.3 (Wong et al., 2018). This focal loss balances between structures that are easy and difficult to segment. Furthermore, we developed the boundary Dice loss to enhance the boundary segmentation accuracy. The Dice loss is effective at increasing the overall overlap between the ground truth and predictions; however, it lacks segmentation accuracy for boundary areas. Thus, to increase the weight of the boundary area, we calculated its Dice loss and added it to the loss for the entire area, which is called hybrid loss (*L*_*hyb*_) in this paper.

$$L_{\text{hyb}}\,\left(g,p\right)=L_{\text{focal}}\,\left(g,p\right)+\lambda \,L_{\text{focal}}\,\left(g-g\,\text{⊖}\,B,p-p\,\text{⊖}\,B\right)$$

In the above equation, we use ⊖ to denote erosion; *B* is the erosion kernel (disk shape with diameter of 7), and λ is the weight for the boundary Dice loss. The boundary was detected through erosion and subtraction, and the Dice loss was calculated from the detected area and added to the whole-area Dice loss. The mixing weight λ was experimentally chosen by evaluating the Dice coefficient of the validation data for each λ in the range of 0.1–0.5; the best performance was obtained for λ = 0.1.

### Aggregation

#### Multi-View Aggregation

Multi-view aggregation combines the predicted results in each orthogonal view, yielding a 3D regularization for errors occurring in 2D plane segmentation (Guha Roy et al., 2019). We trained a separate CNN for each of the three planes: axial, coronal, and sagittal. The predictions of each plane network were aggregated into the final segmentation map. The final segmentation map using multi-view aggregation (*p*_*mv*_) was computed as follows:

$$p_{\text{mv}}\,\left(i\right)=\underset{l}{\arg\max}\left(p_{\text{axi}}\,\left(i,l\right)+p_{\text{cor}}\,\left(i,l\right)+p_{\text{sag}}\,\left(i,l\right)\right)$$

**FIGURE 2:**
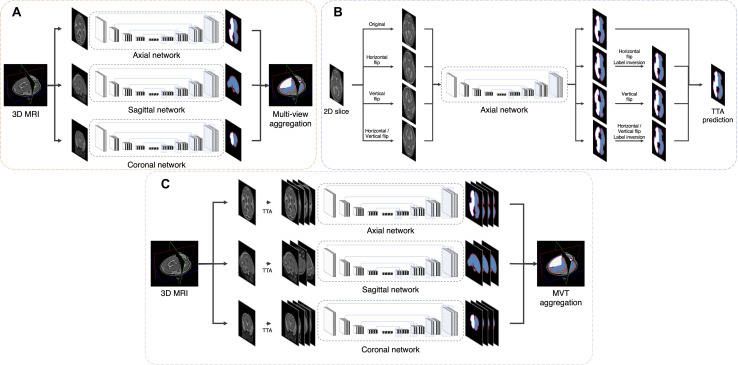
Schematic representation of proposed segmentation procedure. **(A)** Multi-view aggregation combines segmentations from each trained model along three planes: coronal, sagittal, and axial. **(B)** TTA prediction synthesizes multiple segmentations by flip augmentation to generate a final segmentation map. **(C)** To enhance prediction accuracy, MVT aggregation is a combination of multi-view aggregation and TTA.

Here, *p*_axi_(*i*,*l*), *p*_cor_(*i*,*l*), and *p*_sag_(*i*,*l*) are the predicted four-dimensional probability arrays consisting of 3D of the voxel space and one dimension of the labels for axial, coronal, and sagittal planes, respectively. In the *i*-th voxel, the probabilities across the planes are summed and then a label with the highest probability is assigned as the final label. The predicted results for the axial and coronal planes (*p*_*axi*_ and *p*_*cor*_) include 5 labels (background, left inner volume of CP, right inner volume of CP, left CP, and right CP), whereas result for sagittal plane (*p*_*sag*_) contains only 3 labels (background, inner volume of CP, and CP) because there is no information on the left and right hemispheres in 2D sagittal view. Therefore, *p*_*sag*_ of the inner volume of CP is added to both probabilities of left and right inner volume of CP from other planes, and probability of CP is also added to both left and right. Figure 2A illustrates the multi-view aggregation.

#### Test-Time Augmentation

Test-time augmentation has been employed recently to improve the performance of various applications, including segmentation and classification (Matsunaga et al., 2017; Wang et al., 2019). The TTA technique was applied in the testing phase to improve the accuracy by creating various test results and combining these results. Ensemble of multiple prediction results for a single input can reduce prediction errors that may occur in a single prediction. We generated four outputs with artificially augmented inputs: original, horizontal flip, vertical flip, and horizontal/vertical flip (Figure 2B). In the case where slices are inverted left to right, the left and right sides of the output will be inverted from the original state. Therefore, when the left and right sides are inverted, an additional label inversion is applied to switch the left and right labels. For example, the final label map by the axial plane TTA (*p*_*TTA_ axi*_) is computed as follows:

$$p_{\mathrm{sum}\,\_\,\mathrm{axi}}=p_{\text{axi}}+T_{\text{h}}\,\left(p_{\text{axi}}\right)+T_{\text{v}}\,\left(p_{\text{axi}}\right)+T_{\text{hv}}\,\left(p_{\text{axi}}\right)$$

$$p_{\mathrm{TTA}\,\_\,\mathrm{axi}}\,\left(i\right)=\underset{l}{\arg\max}\left(p_{{\text{sum}}_{\text{axi}}}\,\left(i,l\right)\right)$$

Here, *T*_*h*_, *T*_*v*_, and *T*_*hv*_ are the horizontal flip, vertical flip, and horizontal/vertical flip transformation, respectively. This is similar to the multi-view aggregation in terms of combining multiple results, whereas it differs from synthesizing multiple results in one view.

#### MVT Aggregation

MVT aggregation is a combination of 3D information from multi-view aggregation and ensemble of multiple predictions from TTA (Figure 2C). We applied TTA on each plane to obtain multiple results, and aggregated these results from each view to obtain the final result. In this study, the final label value on the *i*-th location [*p*_*MVT*_(*i*)] is computed as follows:

$$p_{\text{MVT}}\,\left(i\right)=\underset{l}{\arg\max}\left(p_{\mathrm{sum}\,\_\,\mathrm{axi}}\,\left(i,l\right)+p_{\mathrm{sum}\,\_\,\mathrm{cor}}\,\left(i,l\right)+p_{\mathrm{sum}\,\_\,\mathrm{sag}}\,\left(i,l\right)\right)$$

Here, *p*_*sum_ axi*_, *p*_*sum_ cor*_, and *p*_*sum_ sag*_ are augmented prediction probability maps obtained from the axial, coronal, and sagittal planes, respectively. By increasing the number of prediction results used in the multi-view, more regularization effects are obtained than in the multi-view aggregation. A total of 11 (4 axial, 4 coronal, and 3 sagittal) prediction results were aggregated to generate the final 3D segmentation label map.

### Training Strategy

Our model was tested with 52 fetuses using ten-fold cross-validation. Stratified sampling was used to match the GA distribution between training folds. 10% of the training samples selected through stratified sampling was used as a validation set. The hybrid loss described above was used for training, and deep learning was optimized using Adam (learning rate = 0.0001) (Kingma and Ba, 2015). For setting the optimal network weights in each fold, we monitored the Dice coefficient in the validation set in every epoch until there is no longer improvement of the Dice coefficient during the last 100 epochs using early stopping function. Then the network weights at the highest Dice coefficient in the validation set were stored as the optimal network. To increase the training dataset, data augmentation was applied. The augmentation parameters were vertical, horizontal, and vertical/horizontal flips. The type of data augmentation applied to the training phase was applied equally to the TTA prediction. For MVT aggregation, three networks of three orthogonal planes were trained. Although the three networks have the same structure, the number of the last outputs from the network of sagittal plane is different from those of axial and coronal planes, because the left and right hemispheres cannot be separated in sagittal plane.

### Evaluation

The automatic segmentation performance was evaluated by the Dice coefficient used to measure the volume overlap and the MSD in order to quantify the boundary accuracy between the ground truth and the prediction segmentation map. The training of the network was based on 2D slices, whereas the proposed method evaluation was conducted in final 3D segmentation result. Furthermore, the CP volume and surface indices were measured and compared between the ground truth and automatically segmented volumes. To calculate the surface index, we adopted surface extraction procedure used in our previous studies (Im et al., 2017; Tarui et al., 2018; Yun et al., 2019, 2020b). Spatial smoothing was performed in the segmented inner volume of the CP using a 1.5 mm full width at half-maximum kernel to minimize noise. Using the smoothed inner volume of the CP, the hemispheric (left and right) triangular surface meshes of the inner CP boundary were automatically extracted by a function “isosurface” in MATLAB 2019b (MathWorks Inc., Natick, MA, United States). The surface models were geometrically smoothed using Freesurfer^1^ to eliminate noise and small geometric changes. We calculated the CP volume based on the automatic segmentation result. Then, the surface area and global mean curvature (GMC) were calculated from the inner CP surface. The surface area was computed based on Voronoi region of each surface mesh vertex (Meyer et al., 2003). Mean curvature was defined as the angular deviation from each vertex (Meyer et al., 2003).

### Statistical Analysis

We evaluated the effect of the loss and aggregation types on the automatic segmentation accuracy in four regions (left inner volume of CP, right inner volume of CP, left CP, and right CP) using the two-way repeated measure analysis of variance (ANOVA). Then, employing the *post hoc* test (Holm–Bonferroni method) for each effect, we determined which loss function and aggregation method performed best. The types of loss functions tested are basic Dice loss and hybrid loss, and the types of aggregation are MVT, multi-view, TTA_*axi*_, TTA_*cor*_, axi, and cor. The axi and cor denote the results obtained using only the original slice without any aggregation. There is no comparison for the sagittal plane since there is no information of the left and right hemispheres. TTA_*axi*_ and TTA_*cor*_ are obtained by applying TTA to the axial and coronal planes, respectively. Multi-view results are obtained from the combination of using only one result without TTA on the three planes, and MVT results from the combination of multiple results by applying TTA on all three planes. The numbers of segmentation aggregations are 11 (MVT), 3 (multi-view), 4 (TTA_*axi*_), 4 (TTA_*cor*_), 1 (axi), and 1 (cor). We used paired t-test to compare the performance between 2D multi-view network and 3D network. For direct comparison between the two networks, the same basic Dice loss was used without TTA. Subsequently, the similarities in the CP volume, surface area, and GMC between manual and automatic segmentation were evaluated using linear regression. Finally, we statistically evaluated whether the segmentation accuracies are associated with data properties, such as the subject age and imaging scanner. We evaluated GA-related changes of the Dice coefficient and MSD using the Pearson correlation analysis. Segmentation accuracies were statistically compared between different MR scanners (47 subjects from Siemens 3T at BCH vs. 5 subjects from Philips 1.5T at TMC) using a permutation test based on random resampling 10,000 times.

## Results

### Effect of Loss Function

**FIGURE 3:**
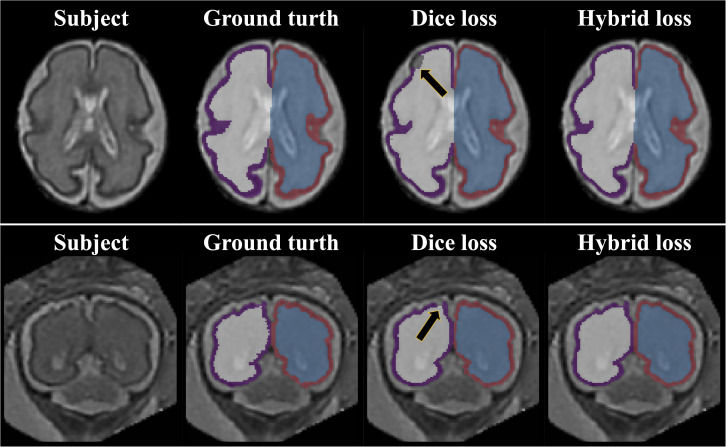
Example of segmentation results with different loss function. The black arrows indicate the errors of segmentation when using the Dice loss. Since the loss for boundary was added, the proposed hybrid loss achieves more accurate segmentation results compared to the Dice loss.

The repeated measure ANOVA test showed no difference between the Dice loss and hybrid loss in the inner volume of CP. However, the hybrid loss had a significantly higher segmentation accuracy (higher Dice coefficient and lower MSD) in the CP compared with the Dice loss (CP Dice coefficient [left, right]: *p* = 0.027, *p* = 0.024; CP MSD: *p* = 0.024, *p* = 0.024). The Dice coefficient and MSD for each loss are shown in Table 1. Figure 3 shows an example of segmentation to verify the effect of hybrid loss.

**TABLE 1:** Statistical comparisons of segmentation performance obtained by different loss functions and aggregation methods.

		**Loss**		**Aggregation**					
		**Hybrid**	**Basic Dice**	**MVT**	**Multi-view**	**TTA_*axi*_**	**TTA_*cor*_**	**axi**	**cor**
Dice	in_L	0.978 ± 0.009	0.978 ± 0.009	0.980 ± 0.008	0.979 ± 0.008^ a^	0.978 ± 0.009^a,b^	0.977 ± 0.009^a,b^	0.977 ± 0.009^a,b,c^	0.976 ± 0.009^a,b,c,d^
	in_R	0.977 ± 0.011	0.977 ± 0.011	0.979 ± 0.011	0.978 ± 0.011^a^	0.977 ± 0.012^a,b^	0.977 ± 0.011^a,b^	0.976 ± 0.011^a,b,c,d^	0.976 ± 0.011^a,b,c,d^
	CP_L	0.899 ± 0.027	0.885 ± 0.048*	0.907 ± 0.027	0.904 ± 0.027^a^	0.897 ± 0.027^a,b^	0.855 ± 0.126^a,b^	0.894 ± 0.026^a,b,c^	0.893 ± 0.029^a,b,c^
	CP_R	0.898 ± 0.031	0.884 ± 0.050*	0.906 ± 0.031	0.902 ± 0.030^a^	0.896 ± 0.032^a,b^	0.896 ± 0.033^a,b^	0.892 ± 0.031^a,b,c,d^	0.851 ± 0.126^a,b^
MSD	in_L	0.293 ± 0.092	0.293 ± 0.095	0.267 ± 0.092	0.277 ± 0.090^a^	0.294 ± 0.097^a,b^	0.299 ± 0.099^a,b^	0.308 ± 0.097^a,b,c^	0.312 ± 0.096^a,b,c,d^
	in_R	0.300 ± 0.112	0.297 ± 0.110	0.271 ± 0.110	0.282 ± 0.107^a^	0.299 ± 0.118^a,b^	0.303 ± 0.116^a,b^	0.318 ± 0.115^a,b,c,d^	0.321 ± 0.108^a,b,c,d^
	CP_L	0.199 ± 0.059	0.544 ± 1.064*	0.188 ± 0.060	0.190 ± 0.058	0.199 ± 0.060^a,b^	1.229 ± 3.178	0.209 ± 0.060^a,b,c^	0.213 ± 0.064^a,b,c^
	CP_R	0.202 ± 0.070	0.551 ± 1.078*	0.186 ± 0.069	0.204 ± 0.077^a^	0.203 ± 0.073^a^	0.205 ± 0.073^a^	0.215 ± 0.072^a,c,d^	1.247 ± 3.192

*Axi and cor denote one original slice result of axial and coronal planes, respectively. TTA_*axi*_ and TTA_*cor*_ aggregate four axial and four coronal view results, respectively. Multi-view aggregates three results (one axial, one coronal, and one sagittal). MVT combines 11 segmentation results (four axial, four coronal, and three sagittal) by applying TTA and multi-view simultaneously. ^∗^Significantly different from hybrid loss; ^*a*^significantly different from MVT; ^*b*^significantly different from multi-view; ^*c*^significantly different from TTA_*axi*_; ^*d*^significantly different from TTA_*cor*_; all significant results: Holm–Bonferroni corrected *p* <  0.05. Data: mean ± standard deviation; in: inner volume of CP; L: left, R: right.*

### Effect of Aggregation Method

**FIGURE 4:**
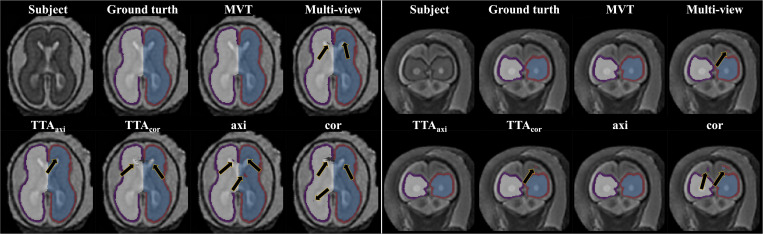
Example of segmentation results with different aggregation methods. The black arrows indicate the errors of segmentation. The proposed MVT method effectively eliminated segmentation errors that remained even after using TTA or multi-view aggregation.

**FIGURE 5:**
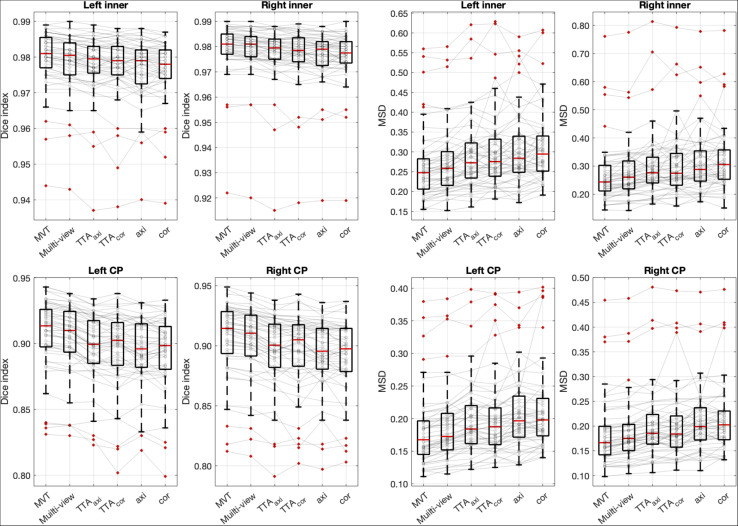
Box plots of segmentation accuracy. The proposed method yields a significantly higher Dice coefficient and lower MSD compared with other methods. The gray line is the connection between the same subjects. *Post hoc* results are listed in Table 1 and Supplementary Tables 1–3.

The Dice coefficient and MSD for each aggregation method are shown in Table 1. In *post hoc* testing, axi and cor showed no statistical difference from each other in all four regions; however, they showed significantly increased accuracy when TTA was applied (TTA_*axi*_ vs. axi and TTA_*cor*_ vs. cor). There was no significant difference between TTA_*axi*_ and TTA_*cor*_. Multi-view aggregation exhibited a better performance than single plane-based TTA. Significantly large differences were found in most regions, except in the MSD of the right CP. Compared with other aggregation methods, the proposed MVT method yielded a significantly higher Dice coefficient in all *post hoc* tests. MVT also showed a significantly lower MSD than other methods in all comparisons except for those with multi-view and TTA_*cor*_ in the left CP and cor in the right CP. All statistical values of the comparisons among aggregation methods are shown in Supplementary Tables 1–3. Figure 4 shows the example of segmentation to verify the effect of each aggregation method. For a visual comparison of the segmentation performance according to the aggregation method, box plots of both evaluation metrics are shown in Figure 5. Additionally, when compared to the 2D multi-view network, the 3D network obtained a significantly lower segmentation accuracy in both the Dice coefficient and MSD (see Table 2).

**TABLE 2:** Statistical comparisons of segmentation performance between 2D network with multi-view aggregation and 3D networks.

		**2D multi-view**	**3D network**	**Paired *t*-test**	
				***t***	***p***
Dice	in_L	0.979 ± 0.008	0.974 ± 0.010	8.352	0.0001
	in_R	0.978 ± 0.011	0.974 ± 0.011	8.563	0.0001
	CP_L	0.904 ± 0.028	0.819 ± 0.223	2.797	0.0073
	CP_R	0.901 ± 0.031	0.881 ± 0.033	12.822	0.0001
MSD	in_L	0.279 ± 0.092	0.369 ± 0.117	−8.067	0.0001
	in_R	0.283 ± 0.108	0.371 ± 0.137	−6.615	0.0001
	CP_L	0.190 ± 0.059	1.875 ± 5.565	−2.134	0.0377
	CP_R	0.217 ± 0.101	0.255 ± 0.081	−3.091	0.0032

*Data, mean ± standard deviation; L, left; R, right.*

### Volume and Surface Index Comparison

**FIGURE 6:**
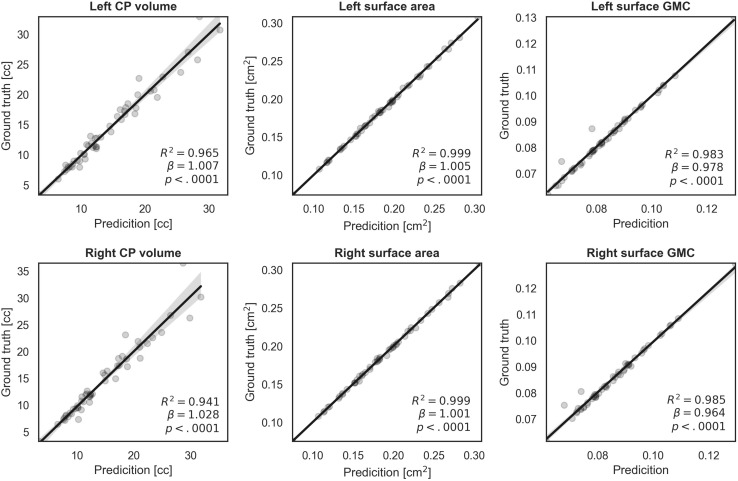
Regression plots of volume, surface area, and surface GMC from ground truth and our automatic segmentation. The fitting result coefficient (β) was very close to unity in all indices in all regions.

We evaluated similarity between the manual and our automatic segmentations in terms of the CP volume, area, and GMC of the inner CP surface. Figure 6 shows the regression results between the indices obtained from the manual and automatic segmentations. The coefficient (β) of the linear regression is close to 1 in all indices, and it is statistically significant (*p* < 0.0001). An *R*^2^ value of 0.94 or more is obtained for all indices. Therefore, the proposed method produced a very similar CP volume and surface indices when compared to manual segmentation.

### Effects of Age and Scanner on Segmentation Performance

**FIGURE 7:**
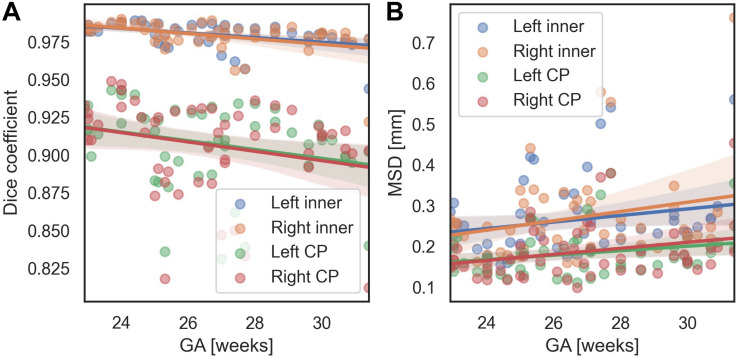
Age-related trends of segmentation accuracy of the proposed method. **(A)** Dice coefficient. **(B)** MSD.

We evaluated the performance of the proposed method with respect to different GA and scanners. In terms of the MSD, for all regions, there were no significant changes of segmentation accuracy by GA (inner volume of CP [left, right]: *p* = 0.113, *p* = 0.063; CP: *p* = 0.089, *p* = 0.055). The Dice coefficient was significantly reduced with GA in the inner volume of CP (left: *p* = 0.001, right: *p* = 0.002). However, the correlations between the Dice coefficient and GA were not statistically significant in the left and right CP (left: *p* = 0.055, right: *p* = 0.073). Figure 7 shows age-related trends of segmentation accuracy.

The accuracies obtained using automatic segmentation did not vary significantly across all regions between the two scanners (inner volume of CP Dice coefficient [left, right]: *p* = 0.402, *p* = 0.406; CP Dice coefficient: *p* = 0.218, *p* = 0.239; inner volume of CP MSD: *p* = 0.603, *p* = 0.628; CP MSD: *p* = 0.384, *p* = 0.357).

## Discussion

We developed a method to segment the CP of the fetal brain with high performance by employing the hybrid loss and MVT. The accuracy of the segmentation results obtained using our proposed method (Dice coefficient > 0.906, MSD < 0.185 mm) was superior to those using previous methods (Bach Cuadra et al., 2009; Habas et al., 2010; Serag et al., 2012; Wright et al., 2014; Khalili et al., 2019). Furthermore, the strong correlations of the volume-based index and surface-based indices between automatic and manual segmentation were found.

### Hybrid Loss Function

We proposed a new hybrid loss to improve the segmentation accuracy at the boundary regions between tissues as well as the overall segmentation performance. Compared with the basic Dice loss, the hybrid loss showed significantly higher Dice coefficient and lower MSD (see Table 1). The proposed loss employed focal Dice loss in order to increase the overall performance, and focal boundary Dice loss in order to increase the boundary accuracy. In the multi-label segmentation problem, the adjustment of the segmentation weight between target labels in the network loss function is one of the primary factors affecting the performance (Sudre et al., 2017). The proposed method adopts a focal structure to adjust the segmentation weight without the need for a weight calculation process. The focal structure created using the logarithmic Dice loss assigns a larger gradient to lower-performance target labels (Wong et al., 2018). As we proposed, our result showed that the hybrid loss was more accurate than the basic Dice loss at the boundary area (Figure 3).

Recently, studies that employ boundary-related loss functions have been conducted (Schmidt and Boykov, 2012; Karimi and Salcudean, 2020). The Hausdorff distance (HD) loss was proposed to include the surface distance in the loss function (Karimi and Salcudean, 2020). However, the calculation process is complicated, and the weight compensation is difficult as the range of values of the Dice and boundary loss vary. In this study, we proposed a morphological erosion-based boundary Dice loss which is simple and similar to the whole-area Dice loss, and the weight adjustment is straightforward as the range is the same as the whole-area Dice loss. An additional experiment was conducted to compare the segmentation performance between the HD loss (focal Dice loss + HD loss) and the hybrid loss proposed in this paper. There was no statistical difference between the two loss functions (paired *t*-test, CP Dice coefficient [left, right]: *p* =  0.686, *p* =  0.544; CP MSD: *p* =  0.398, *p* =  0.243). The proposed method has an advantage because it not only requires much simpler computation and weight control compared to the previous study (Karimi and Salcudean, 2020), but also shows a high segmentation performance.

### MVT Aggregation

We propose the MVT aggregation, which combines multi-view aggregation and TTA. Compared to other aggregation methods, the proposed method showed significant increases in the Dice coefficient exhibited in all regions. The MSD significantly decreased in all regions except for the left CP of multi-view, the left CP of TTA_*axi*_, and the right CP of cor. Our deep learning network did not fully utilize the 3D information of MRI as it was trained based on 2D slices. Therefore, to correct 2D results using 3D information, a multi-view aggregation was adopted, which synthesizes the results from networks of three orthogonal planes to generate a final 3D segmentation result. TTA was applied to improve the accuracy using various predicted segmentation maps. TTA improves the prediction accuracy by applying data augmentation to obtain multiple prediction results and ensemble them. As a result of the evaluation, we found that higher accuracies were obtained with a larger number of segmentation results (Table 1 and Supplementary Tables 1–3). TTA results (TTA_*axi*_ and TTA_*cor*_) showed higher accuracies than those of one slice (axi and cor) (Table 1 and Supplementary Table 1). The results demonstrate that multi-prediction by TTA can reduce errors that may occur in single prediction (axi and cor). Notably, multi-view results were more accurate than TTA (Table 1 and Supplementary Table 2). For the final segmentation map, multi-view aggregation was corrected using three results from three planes, and it was more accurate than the TTA corrected with four results from one plane. This result indicates that 3D information from multi-view aggregation is more helpful for precise segmentation than the ensemble of the result using TTA. Also, multi-view aggregation outperformed the 3D network (Table 2). Although multi-view aggregation approach is based on a 2D network, it can reflect 3D information and utilize more training data, which may result in better segmentation performance compared to 3D network. MVT proposed in this study combines four results in the axial plane, four results in the coronal plane, and three results in the sagittal plane to finally produce the final segmentation with 11 prediction results. Therefore, the ensemble of multiple predictions using TTA was obtained, and at the same time, to generate more accurate segmentation results, the regularization of 3D information using multi-view aggregation was incorporated. The comparison of MVT with other approaches is shown in Table 1 (Supplementary Table 3). Figure 4 shows that as the level of the synthesis increases, the segmentation error decreases. It is shown that the error caused by prediction using only one slice can be corrected by TTA or multi-view, but more effectively by MVT.

### Measurement of Volume- and Surface-Based Indices Using Automatic Segmentation

The accurate segmentation of brain regions is a fundamental step for the further analysis of brain morphometry using volume- and surface-based indices. The indices obtained from our segmentation method showed high correlations with the corresponding indices obtained from ground truth. When the CP volume is small, accurate results were obtained, whereas when the volume of the CP increased (>20 cc), the fitting accuracy decreased. This occurs because as the fetus grows and the brain size increases, the CP quickly becomes more complex and folded, increasing the difficulty of automatic segmentation. However, the actual average prediction errors remained low at values as small as 1.714 cc for the left CP and 2.308 cc for the right CP. Compared to the CP volume, regression models of surface indices showed higher correlations in the whole GA range between manual and automatic segmentation. Thus, our findings demonstrate that the proposed automatic segmentation method is reliable for further volume- and surface-based analyses.

### Gestational Age and Scanner Effects on CP Segmentation

We used the Dice coefficient, MSD, and volume- and surface-based indices to evaluate the segmentation accuracy. Among them, in the fitting result for the CP volume, the accuracy of fitting tends to decrease as the volume of the CP increases. This trend is assumed to be related to the effect of the GA on the segmentation accuracy. The accuracy of CP segmentation exhibited a decreasing trend with an increase in GA, which is likely to result from the increasing complexity of the CP folding. However, the relationship between the GA and CP segmentation accuracy was not statistically significant. Upon measuring the accuracy for fetuses older than GA 30 weeks, the average Dice coefficient and MSD were 0.967 and 0.323 mm, respectively, for the inner volume of CP, and 0.891 and 0.220 mm, respectively, for the CP. Hence, the proposed method demonstrated a high level of segmentation performance even in older fetuses. Additionally, Supplementary Figure 1 shows local segmentation errors with different GA group. We divided the GA into three groups (22.9–25.3 [*n* = 19], 25.3–27.5 [17], 27.5–31.4 [16]), and showed examples of the segmentation errors for the subjects having the maximum, median, and minimum CP Dice coefficient in each GA group.

We performed permutation tests to verify whether there is any significant difference in the segmentation performance depending on the scanner. No statistical difference was found between scanners for all metrics, indicating that our results were not biased by the scanner effect.

### Comparison With Other Methods

We propose a deep learning network for CP segmentation using MR images obtained from 52 fetuses. The proposed method obtained a Dice coefficient of 0.907 ± 0.027 and 0.906 ± 0.031, and an MSD of 0.182 ± 0.058 mm and 0.185 ± 0.069 mm for the left and right CP, respectively, using hybrid loss and MVT. Compared with other methods, we used a larger sample of the fetal dataset and varied the number of labels for segmentation. Therefore, it is difficult to compare the methods directly. Our proposed segmentation method was compared directly with a recent fetal CP segmentation deep learning model and indirectly with previous methods that used the EM algorithm and atlas-based segmentation. To the best of our knowledge, only two MRI studies and one ultrasound study have proposed the fetal CP segmentation method using deep learning (Khalili et al., 2019; Dou et al., 2020; Wyburd et al., 2020). Among them, our method was directly compared to one peer-reviewed study (Khalili et al., 2019). The authors applied a 2D U-Net with basic Dice loss to coronal MRI slices obtained from 12 fetuses, and a Dice coefficient of 0.835 and MSD of 0.307 mm were obtained for the CP volume (Khalili et al., 2019). When compared with our proposed deep learning model, the structure of the model was the same, but the loss function used for training was different and the MVT approach was not used. Therefore, of the results in this paper, the result obtained using basic Dice loss in the network for coronal slices can be considered to result from the method of the prior study (CP Dice coefficient [left, right] = 0.894 ± 0.030, 0.811 ± 0.251; CP MSD = 0.212 ± 0.064 mm, 2.277 ± 6.388 mm). The proposed method showed a significantly higher segmentation accuracy using hybrid loss and MVT compared to the prior deep learning method (Khalili et al., 2019) (CP Dice coefficient [left, right]: *p* <  0.0001, *p* =  0.009; CP MSD : *p* <  0.0001, *p* =  0.022). The results obtained by the EM algorithm were as follows: (Bach Cuadra et al., 2009): 4 subjects; 29–32 weeks GA; Dice coefficient = 0.63 ± 0.04; MSD = 0.70 ± 0.08 mm, (Habas et al., 2010): 14 subjects; 20.57–22.86 weeks GA; Dice coefficient = 0.82 ± 0.02, (Wright et al., 2014): 16 subjects; 22.4–36.4 weeks GA; MSD = 0.86 ± 0.14 mm. The atlas-based segmentation method reported a Dice coefficient of 0.84 ± 0.06 for CP using MRI data from 15 fetuses (21.7–38.7 weeks GA) (Serag et al., 2012). Detailed results are shown in Table 3. Our method shows a better performance in terms of both the Dice coefficient and MSD when directly or indirectly compared to previous methods. The GA range of fetal subjects included in our study is narrower compared to some of the previous studies (Serag et al., 2012; Wright et al., 2014), which may result in higher accuracy as the older fetal brain MRI scans with complex folding are more difficult for CP segmentation. However, compared with the results obtained in our study, those studies utilized very few fetal MRI scans (≤16) and showed considerable differences in the Dice coefficient and MSD. Moreover, we found no significant correlations between the GA and CP segmentation accuracy, and obtained high accuracies even for fetuses over 30 weeks GA, as described above. Therefore, the narrow GA range in our study was not a bias causing the high accuracy. The previous deep learning study employed the basic Dice loss in multi-label segmentation, and showed relatively poor performance in small volume labels (Sudre et al., 2017; Wong et al., 2018). Although the authors applied several augmentation methods to increase the amount of training data in deep learning, they did not include the correction achieved by multiple predictions. The higher accuracy obtained in our method may be attributed to the inclusion of a loss function suitable for multi-label segmentation and correction by multiple predictions using MVT. The relatively low performance of the EM algorithm and atlas-based segmentation may be due to the registration quality as the brain template created by combining multiple images is blurred compared to individual images. It is not easy to obtain an accurate registration of the brain template to a target subject image, even with non-linear transformation. Furthermore, the partial volume effect of the CP boundary owing to the limited fetal MRI resolution and motion decreases the accuracy with which the likelihood probability of the EM algorithm and the registration accuracy of the atlas-based method can be estimated. The proposed method used only linear registration to unify the size of input images. Unlike previous methods, deep learning is free of registration effects because it does not need to accurately match any prior information. Furthermore, the inaccuracy that results from the partial volume effect may also be sufficiently trained by deep learning to enable a similar segmentation, as is possible with the ground truth. The proposed deep learning network exhibits a higher segmentation performance using hybrid loss and MVT than other methods.

**TABLE 3:** Cortical plate (CP) segmentation performance of the proposed method and other methods.

		Deep learning (direct)		EM (indirect)			Atlas-based (indirect)
	
				
		Proposed	Khalili et al., 2019	Bach Cuadra et al., 2009	Habas et al., 2010	Wright et al., 2014	Serag et al., 2012
No. subject (GA range)		52 (22.9–31.4)	52 (22.9–31.4)	4 (29–32)	14 (20.6–22.9)	16 (22.4–36.4)	15 (21.7–38.7)
Dice	CP_L	**0.907 ± 0.027**	0.894 ± 0.030	−	−	−	−
	CP_R	**0.906 ± 0.031**	0.811 ± 0.251	−	−	−	−
	CP	**0.907 ± 0.029**	0.852 ± 0.141	0.625 ± 0.038	0.82 ± 0.02	−	0.84 ± 0.06
MSD	CP_L	**0.182 ± 0.058**	0.212 ± 0.064	−	−	−	−
	CP_R	**0.185 ± 0.069**	2.277 ± 6.388	−	−	−−	−
	CP	**0.184 ± 0.063**	1.245 ± 3.226	0.697 ± 0.079	−	0.864 ± 0.141	−

*For direct comparison with the previous deep learning method of (Khalili et al., 2019), we implemented their method and applied it to our dataset. The Dice coefficient and MSD of EM and atlas-based methods were taken from their published papers for indirect comparison. The proposed method shows a higher Dice coefficient and a lower MSD when compared to previous studies either directly or indirectly. Data, mean ± standard deviation; L, left; R, right. Bold values indicate the results of our proposed method that show the best performance.*

### Limitations

Despite the accurate CP segmentation with MVT and hybrid loss, there are some limitations to the proposed method. First, because the folding pattern of the fetal brain changes dynamically and becomes more complex as gestation progresses, a decreasing trend was observed in the CP segmentation accuracy with age although it was not statistically significant. Therefore, to improve the segmentation accuracy, it is necessary to include a larger number of fetuses above 30 weeks GA. Second, the proposed model did not include cerebrospinal fluid (CSF). In particular, the segmentation of deep sulcal CSF is essential for precise outer CP surface extraction, which enables the further analysis of cortical measures, such as cortical thickness. However, because of the limited resolution of fetal brain MRI scans and the partial volume effect of CSF in narrow deep sulcal regions, the manual segmentation of CSF in these regions is highly challenging. Although CSF segmentation was included in previous studies (Wright et al., 2014; Khalili et al., 2019), it has not been designed to extract deep sulcal CSF. In future studies, we will carefully delineate fetal CSF regions and train them to develop an automatic method for CSF segmentation.

## Conclusion

The proposed method segments the fetal CP providing highly accurate measurements of CP volume and the highly accurate surface reconstruction of the CP. The hybrid loss and MVT show a significant increase in accuracy compared to the basic Dice loss and other aggregation methods. Although most of our comparisons were performed indirectly, the proposed method showed better fetal CP segmentation performance than other methods. Likewise, the comparisons of CP volume and surface indices between prediction and ground truth showed high similarity. Our results indicate that our proposed automatic segmentation method is useful for performing an accurate quantitative cortical structural analysis in the human fetal brain. The developed automatic segmentation is more reproducible than manual segmentation as it is not affected by inter- and intra-rater variability, and it has a short computation time.

## Data Availability Statement

The datasets generated for this study are not readily available because the fetal MRIs used in this study was not available publically. Requests to access the datasets should be directed to KI, kiho.im@childrens.harvard.edu.

## Ethics Statement

The studies involving human participants were reviewed and approved by Institutional Review Boards at the Boston Children’s Hospital (BCH) and Tufts Medical Center (TMC). Written informed consent to participate in this study was provided by the participants’ legal guardian/next of kin.

## Author Contributions

KI, HJY, and J-ML designed the main idea and directed the overall analysis. JH and HJY developed the algorithm and carried out the data processing and experiments. GP, SK, CL, LS, TT, CR, CO, and PG assisted with the data collection and result interpretation. JH, HJY, KI, and J-ML wrote the manuscript with input from all authors.

## Conflict of Interest

The authors declare that the research was conducted in the absence of any commercial or financial relationships that could be construed as a potential conflict of interest.

## Footnotes

1 https://surfer.nmr.mgh.harvard.edu
