# Supplementary material for: Fetal Cortical Plate Segmentation Using Fully Convolutional Networks With Multiple Plane Aggregation
Source: Front Neurosci. 2020 Dec 2;14:591683. doi: 10.3389/fnins.2020.591683 (PMC7738480; doi:10.3389/fnins.2020.591683)
Supplement: Supplementary file 1 [file Table_1.DOCX]

Supplementary Material

# Supplementary Figures and Tables

## Supplementary Tables

**Supplementary Table 1.** Post-hoc results among different TTA aggregations. Bold values indicate the statistical significance.

|  |  | TTA_axi_ – TTA_cor_ | |  | TTA_axi_ – axi | |  | TTA_axi_ – cor | |  | | TTA_cor_ – axi | | |  | | TTA_cor_ – cor | | |  | | axi – cor | | |  |
| --- | --- | --- | --- | --- | --- | --- | --- | --- | --- | --- | --- | --- | --- | --- | --- | --- | --- | --- | --- | --- | --- | --- | --- | --- | --- |
|  | | *t* | *p* |  | *t* | *p* |  | *t* | *p* | |  | | *t* | *p* | |  | | *t* | *p* | |  | | *t* | *p* | |
| **Dice** | in_L | 0.993 | .6512 |  | 4.616 | **.0001** |  | 3.324 | **.0066** | |  | | 1.581 | .3601 | |  | | 4.651 | **.0001** | |  | | 0.724 | .6512 | |
|  | in_R | 0.748 | .9155 |  | 6.010 | **.0001** |  | 3.333 | **.0064** | |  | | 2.886 | **.0171** | |  | | 5.099 | **.0000** | |  | | 0.351 | .9155 | |
|  | CP_L | 2.406 | .0792 |  | 4.936 | **.0001** |  | 3.961 | **.0016** | |  | | –2.241 | .0881 | |  | | –2.157 | .0881 | |  | | 1.022 | .3114 | |
|  | CP_R | 0.127 | .8992 |  | 7.849 | **.0001** |  | 2.567 | .0529 | |  | | 3.201 | **.0165** | |  | | 2.548 | .0529 | |  | | 2.346 | .0529 | |
| **MSD** | in_L | –1.198 | .4732 |  | –4.282 | **.0004** |  | –3.555 | **.0033** | |  | | –1.959 | .1669 | |  | | –4.377 | **.0004** | |  | | –0.870 | .4732 | |
|  | in_R | –0.947 | .6958 |  | –4.535 | **.0002** |  | –3.783 | **.0016** | |  | | –3.114 | **.0091** | |  | | –5.547 | **.0001** | |  | | –0.709 | .6958 | |
|  | CP_L | –2.334 | .1509 |  | –6.952 | **.0001** |  | –4.679 | **.0002** | |  | | 2.312 | .1509 | |  | | 2.301 | .1509 | |  | | –1.270 | .4200 | |
|  | CP_R | –0.820 | 1.000 |  | –8.062 | **.0001** |  | –2.358 | .1827 | |  | | –3.421 | **.0124** | |  | | –2.355 | .1827 | |  | | –2.331 | .1827 | |

**Supplementary Table 2.** Post-hoc comparison of multi-view aggregation with TTA. Bold values indicate the statistical significance.

|  |  | Multi-view –  TTA_axi_ | |  | Multi-view –  TTA_cor_ | |  | Multi-view –  axi | |  | Multi-view –  cor | |
| --- | --- | --- | --- | --- | --- | --- | --- | --- | --- | --- | --- | --- |
|  | | *t* | *p* |  | *t* | *p* |  | *t* | *p* |  | *t* | *p* |
| **Dice** | in_L | 4.978 | **.0001** |  | 7.025 | **.0001** |  | 7.278 | **.0001** |  | 10.865 | **.0001** |
|  | in_R | 4.690 | **.0001** |  | 5.706 | **.0001** |  | 9.565 | **.0001** |  | 9.425 | **.0001** |
|  | CP_L | 9.058 | **.0001** |  | 2.782 | **.0378** |  | 12.591 | **.0001** |  | 13.336 | **.0001** |
|  | CP_R | 8.125 | **.0001** |  | 6.653 | **.0001** |  | 13.072 | **.0001** |  | 2.960 | **.0233** |
| **MSD** | in_L | –5.166 | **.0001** |  | –7.707 | **.0001** |  | –6.855 | **.0001** |  | –9.672 | **.0001** |
|  | in_R | –4.764 | **.0001** |  | –6.048 | **.0001** |  | –8.462 | **.0001** |  | –8.973 | **.0001** |
|  | CP_L | –6.770 | **.0001** |  | –2.354 | .1509 |  | –10.972 | **.0001** |  | –8.537 | **.0001** |
|  | CP_R | 0.308 | 1.000 |  | –0.077 | 1.000 |  | –1.912 | .2460 |  | –2.381 | .1827 |

**Supplementary Table 3.** Post-hoc comparison of MVT to other aggregation methods. Bold values indicate the statistical significance.

|  |  | MVT –  Multi-view | |  | MVT –  TTA_axi_ | |  | MVT –  TTA_cor_ | |  | MVT –  axi | |  | MVT –  cor | |
| --- | --- | --- | --- | --- | --- | --- | --- | --- | --- | --- | --- | --- | --- | --- | --- |
|  | | *t* | *p* |  | *t* | *p* |  | *t* | *p* |  | *t* | *p* |  | *t* | *p* |
| **Dice** | in_L | 7.665 | **.0001** |  | 7.656 | **.0001** |  | 10.077 | **.0001** |  | 9.169 | **.0001** |  | 13.452 | **.0001** |
|  | in_R | 6.855 | **.0001** |  | 7.892 | **.0001** |  | 9.349 | **.0001** |  | 12.865 | **.0001** |  | 11.824 | **.0001** |
|  | CP_L | 7.696 | **.0001** |  | 13.641 | **.0001** |  | 2.960 | **.0280** |  | 15.375 | **.0001** |  | 16.142 | **.0001** |
|  | CP_R | 9.985 | **.0001** |  | 12.791 | **.0001** |  | 11.240 | **.0001** |  | 16.845 | **.0001** |  | 3.150 | **.0165** |
| **MSD** | in_L | –6.233 | **.0001** |  | –8.075 | **.0001** |  | –9.586 | **.0001** |  | –7.972 | **.0001** |  | –10.179 | **.0001** |
|  | in_R | –5.556 | **.0001** |  | –8.525 | **.0001** |  | –8.182 | **.0001** |  | –9.284 | **.0001** |  | –9.065 | **.0001** |
|  | CP_L | –0.618 | .5395 |  | –4.100 | **.0012** |  | –2.371 | .1509 |  | –7.070 | **.0001** |  | –6.236 | **.0001** |
|  | CP_R | –3.542 | **.0095** |  | –10.849 | **.0001** |  | –7.892 | **.0001** |  | –13.511 | **.0001** |  | –2.396 | .1827 |

**Supplementary Figure 1.** Example of segmentation error maps due to development of fetal brain (Blue: false positive, Red: false negative).
